# Contrariwise obesity through organic food consumption in Malaysia: a signaling theory perspective

**DOI:** 10.1186/s12889-021-12480-3

**Published:** 2022-01-14

**Authors:** Zulhamri Abdullah, K. Y. S. Putri, Syed Hassan Raza, S. Bekti Istiyanto

**Affiliations:** 1 Department of Communication, Faculty of Modern Language and Communication, Universiti Putra Malaysia, Selangor, Malaysia; 2 Program Studi Ilmu Komunikasi, Fakultas Ilmu Sosial, Universitas Negeri Jakarta, Jakarta, Indonesia; 3 Department of Communication Studies, Bahauddin Zakariya University, Multan, Pakistan; 4 Universitas Jenderal Soedirman, Purwokerto, Indonesia

**Keywords:** Healthy lifestyle, Obesity, Theory of planned behavior, Purchase intention, Environmental concern, Consumer innovativeness

## Abstract

**Background: the context and purpose of the study:**

Unhealthy food consumption has raised an alarming situation of obesity among Asian nations and posing serious threats to human health. Recent studies have acknowledged that organic food consumption has been contrariwise associated with obesity. The consumption of healthy food has received research attention in social marketing and several antecedents and consequences have been identified. However, to date, there is a void in literature that how social, individual, and marketing elements together tradeoff in predicting a healthy lifestyle. Thus, the current investigation unfolds the antecedents of healthy foods’ adoption in Asia by integrating the brand signaling and theory of planned behavior.

**Methods:**

The data of 241 respondents were collected from selected social media Facebook communities through a survey using assessed 42 questions. For this purpose, participants’ Facebook accounts were selected from the online healthy communities such as ‘*Diet Suku Suku Separuh’* (469,000 followers), ‘Hiking, and Camping around Malaysia’ (351,200 followers), and ‘Healthy Malaysia’ (332 followers). The enumerator also engaged with the online community by liking posts and following health accounts.

**Results:**

The data was analyzed using PLS (SEM) approach, the outcomes of hypotheses revealed interesting information that health consciousness not significantly predicts the purchase intention of healthy food. All antecedents were significant contributors to the prediction of foods’ purchase intentions in this study. However, the findings indicated that no positive relationship exists between brand image identifications and brand credibility identifications, and healthy foods’ purchase intentions identifications. The findings also indicated that no positive relationship exists between health consciousness identifications and healthy foods’ purchase intentions identifications.

**Conclusions: (summary and potential implications):**

Owing to the perilous increase in obesity among the general public in Asia. This study reinforced the factor that can help in the adoption of a healthy lifestyle. The study validated that a healthy lifestyle is reliant on the consumers’ health consciousness, environmental concern, and innovativeness through motivating the consumers’ healthy foods’ purchase intentions. Surprisingly, the results highlighted that respondents have not identified brand image and credibility as an antecedent of purchase intention. Given that organic food brands are somewhat new in Asian markets and therefore, brands must endure crisis marketing practices to improve their brand recognition. Therefore, policymakers must facilitate the food promotional activities that are critical to enhancing the perceived benefits of organic food to combat issues like obesity. This paper offers a foundation for future empirical investigations in Asia and various stakeholders on how to promote a healthy lifestyle in Asia. Specifically, the results will help policymakers to offer positive policies and procedures for the improvement of a healthy lifestyle through the understanding of the antecedents and consequences of health-conscious consumers’ healthy foods’ purchase intentions.

## Introduction

**Fig. 1:**
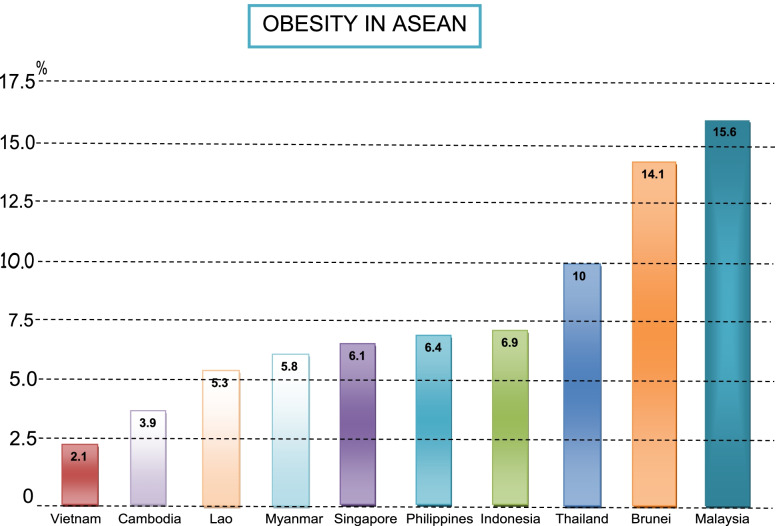
Obesity in Asia, Source: World Population Review, 2020

Obesity and dietary patterns have become distressing public health issues. Recent global death rates from diseases associated with obesity have been increased ten-fold during previous decades [1]. In 2016, 39% of adult men and 40% of women, about 1.9 billion people aged 18 and over, were overweight [2]. While 11% of adult men and 15% of women, accounting for almost 500 million, were obese globally [2]. Obesity has shown a noticeable upsurge over the previous three decades [3]. Unhealthy diets and physical inactivity cause both overweight and obesity among people [4]. The ongoing transition in dietary habits of the people exemplified is troublesome. Currently, the nutritional patterns of people across the world contain an insufficient amount of requisite nutrients and abundant with carbohydrates leading to obesity [5]. For this reason, global trends in obesity and its associated diseases such as diabetes and hypothyroidism are rapidly increasing [4]. A similar alarming upsurge trend of obesity has been observed during the last two decades in South-East Asian countries. According to the obesity ranking in the Asian region, Malaysia was listed as the nation with greatest extent of obesity [6] (Refer to Fig. 1). According to the WHO, Malaysia with a nearly 50% obesity rate, is among the fattest nation in Asia [3]. The WHO data indicates that 44.4% of adults have more than 25 kg Body Mass Index (BMI) and are overweight [7]. Owing to the higher obesity rate Malaysia is spending around 20% of the healthcare budget on the diseases associated with obesity [8].

To overcome this prevailing situation of obesity, policymakers of Malaysia took the initiative to tackle these prevalent public health issues and formulated the “National Plan of Action for Nutrition in Malaysia (NPANM I) (1996 to 2000)”. The purpose of this program was to chalk out strategies on how to minimize obesity through food policy, awareness, and promotional campaigns [7]. Undesirably, these strategies remained unsuccessful and during the period, the frequency of obesity and overweight rose to 177% [9]. Healthy and sustainable organic products using various technologies have been introduced in emerging markets [10]. Healthy product development is the degree to which ecological issues should be recognized developing of such products [11]. According to the U.S. Department of Agriculture, the word ‘organic’ refers to any food produced using accepted farming techniques that conserve biodiversity, did not harm natural resources, and applied only accepted substances [12]. Thus, organic food is made devoid of using conservative pesticides [13]. “In terms of food that comes from living animals – meat, eggs, and dairy products, the animal must not be fed antibiotics or growth hormones” [13]. Any food that is term organic in nature must be environmentally safe [14], produced using a technique that does not require modern artificial inputs including chemical fertilizers, pesticides and include organisms that are genetically modified [12] not processed with irradiation, not processed using chemical food additives or/and industrial solvents [14].

Therefore, recent food technologies such as organic foods have provided healthy food choices to people [15]. For example, emerging consumption trends of organic food intake have been suggested as a remedy to vanquish obesity and overweight. Recent research provides evidence that organic food consumption has an inverse effect on obesity [16]. However, past research has identified several factors involved in the adoption of a healthy lifestyle [10, 17, 18]. For example, analysis revealed that health-conscious customers demand sustainable products, including organic products with sustainable characteristics [18]. Therefore, research affirmed that health-conscious customers’ choices of organic foods are influenced by their perceived health benefits of organic foods. These consumers utilize health compare to organic foods with drugs that cause side effects to the consumers [19–23]. In contrast, studies reported that consumers in Asian markets have a lower health-conscious extent than the Western nation. Therefore, they lack trust in the brands promoting these products due to higher uncertainties about safety [12]. The Asian consumers are thereby, more conscious of purchasing organic food products, quality, and performance [21, 22, 24]. Regardless of marketing and promotion strategies in practice, people are reluctant to adopt such a healthy lifestyle in Asia. Consequently, a pertinent question is about the public understanding of healthy food intake and why they do not value the food choices that can benefit their health and the environment. Thus, this public health issue remains unaddressed, and research-driven promotional campaigns are required. In the past, few studies have been carried out to tap the factors involving in involved in purchasing and adopting of healthy products in Asian countries [25], including Malaysia. Therefore, the main purpose of our paper is to investigate the effect of health consciousness, environmental concern, and innovativeness on health-conscious consumers’ food purchase intentions and consequently improve their healthy lifestyle. This study offers empirical evidence that could help policymakers and Malaysian citizens increase the use and purchase of organic food products, undoubtedly improving their healthy lifestyles.

## Literature review

### A conceptual framework and hypotheses development

**Fig. 2:**
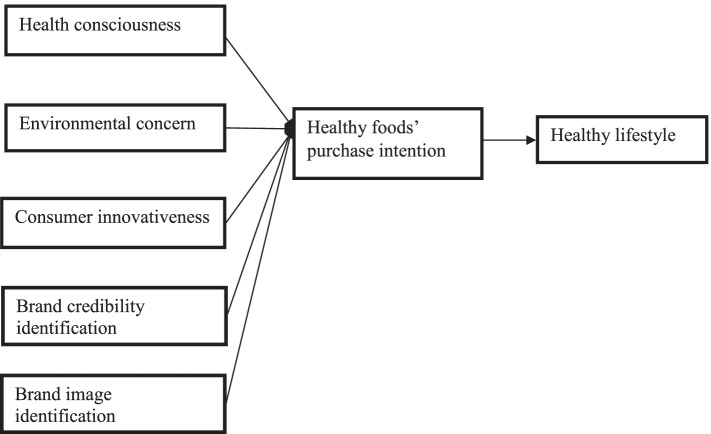
Proposed Conceptual Framework

In this paper, the TPB model was used as a theoretical foundation to elucidate organic food purchase intention among health-conscious consumers in Malaysia [26]. The TPB was developed in 1991 by Ajzen from the former [27] theory of reasoned action. The TPB states that a particular behavior of an individual is drive by an individual intention to perform that action [26]. The intention mirrors individual motivations and cognitive preparation for performing the behavior. The intention is determined by three vital factors: subjective norm, attitude, and perceived behavioral control [26]. Attitude mirrors the negative or positive assessment of the individual behavioral consequences [28]. Subjective norm reflects how the individual perceived social pressure influence the performance of a particular behavior. In contrast, perceived behavioral control is associated with the person’s perception of his/her capability to perform that specific behavior. The TPB variables were proved to be important predictors of various food choices including organic foods purchase [19–22]. Figure 2 depicts that the three fundamental factors of individual behavior: subjective norm, attitude, and perceived behavioral control are fundamental antecedents of individual intention to perform a particular behavioral intention, and consequently mediate these antecedents with actual individual behavior [29].

The present paper aims to investigate factors that influence healthy conscious consumers’ purchase intention of organic food; TPB is used as the foundation of the current study because of its robustness and wide applications in understanding consumer purchase decisions in various studies [20–22]. For instance, if health-conscious consumers have a positive attitude to engage in a particular behavior (e.g., purchasing organic food), and believe the approval and support of friends and family to buying organic food, then health-conscious consumers are more expected to perform the actual behavior of the purchase [30]. Concerning the TPB predictive power in the existing literature especially in the context of organic food purchase, the TPB explained a 61% variance of the individuals’ intention to purchase organic food [31] and 83% in the study of Thøgersen [32]. TPB also explained 82% variance of the individuals’ intention to purchase organic food in the study of Tarkiainen and Sundqvist [33]. Dowd and Burke [34] also established the robustness of this model in investigating organic food purchases explaining 62% variation in intention.

Within the framework of organic food purchase intention, nevertheless, two major types of investigations can be differentiated: studies from the marketing context that are mainly paying attention to the comprehension of consumers’ motivations and those largely paying attention from industrial ecology that is typically concerned in the impact of individual consumer’s behaviors [35, 36]. While the subsequent approach centered on the outcome of individual behavior, the earlier examines the motivations behind the behaviors. Therefore, following the first approach, a wide variety of studies within the organic food purchase literature has used TPB [37] as the theoretical foundation for examining the various factors that make healthy conscious consumers’ behavioral intentions towards purchasing organic food products [20–22].

Prior studies point out that health-conscious consumers are more expected to pay a high premium for the quality of organic foods products [19–23]. Given the fast and accelerated buying and sales of organic foods products, understanding the important antecedents that influence health-conscious consumers’ organic food product purchase intentions is necessary for producers of organic products, marketing specialists, green restaurateurs, suppliers, and policymakers to apply profitable strategies. Reviewed of the earlier literature reported that health-conscious consumers’ motives of purchasing organic food include wholesome lifestyle, health concerns, concerns of the environment, food safety concerns, and protection of animal welfare. Very limited studies investigated the influence of health consciousness, environmental concern, and innovativeness on healthy conscious consumers’ food purchase intentions. Using TPB and signaling theory, this study incorporated health consciousness, ecological concern, and innovativeness as antecedents of purchase intentions and added a healthy lifestyle as the outcome of health-conscious consumers’ purchase intention in Asia. The conceptual framework is in Fig. 2.

### Purchase intention

In psychological research, human behaviors are generally considered measurable and observable [38]. The psychologies categorize human behaviors to include individual attitudes, his/her thoughts, beliefs, perceptions, and intentions [38]. The word “intention” is defined differently across studies by various authors. Ajzen [39] offered the most generally accepted definition of behavioral intention as “indications of a person’s readiness to perform a behavior” (p. 1122). Interestingly, the main focus of this paper is to study the antecedents of health-conscious consumers’ intention to purchase organic food products in Asia. A range of empirical studies recognized that consumers’ behavioral intentions to purchase a particular product are the primary determinant of their actual behavior [24]. For example, Ajzen [29] reported that the most vital factors that decide consumers’ actual purchase of a particular product are the consumers’ intention. Consumers’ behavioral intentions variable is widely researched to understand various factors that influence consumers’ purchase of organic products [40].

In the Ajzen TPB model, the intention the consumers’ willingness, effort, and plan toward purchasing a product [20, 21, 29]. In other words, consumers’ intention specify their maximum likelihood to engage in a particular action soon [29]. Spears and Singh [41] refer to consumers’ purchase intention as “an individual’s conscious plan to make an effort to purchase a product”. Organic food purchase intention is defined as the health-conscious consumers’ readiness and willingness to purchase organic food products articulated by the consumers for the friendly environment and health benefits [40]. In other words, consumers who are willing to purchase organic products are mainly concerned about the products’ ecological quality and the consequences of the environment related to their purchasing decision [20–22].

The organic food products industry is growing rapidly in Malaysia, as demand has increased because of health-conscious consumers’ increasing awareness regarding the health benefits of consuming organic food [35, 36]. Since health-conscious consumers consider eating organic food advantageous to their health, their positive attitudes have drastically influenced their purchase intention [22, 36].

### Health consciousness

Due to the pandemic crisis, it is essential to conceptualize e-health literacy to understand better health consciousness among consumers [42]. Chen [43] opined that health factors such as health consciousness should be considered while purchasing organic food. This is because one of the major significant for consumers to buy organic food is their health [43]. Likewise, the consumers’ motivation towards health-related food such as organic food is to prevent them from disease or improve their health and thus, is one of the important factors for purchase behavior and intentions [25, 44]. Health consciousness refers to individuals’ readiness to take on healthy actions or behavior that will improve their health rather than taking on the general unhealthy consumption patterns [43, 44]. In other words, health consciousness refers to the motivational elements that encourage consumers to carry out health actions [45]. Consumers who have health-conscious in purchase decisions are usually aware and concerned regarding their health and wellness. Additionally, these consumers are self-conscious about their health and are motivated on any purchase decision to enhance and/or maintain their health, healthy lifestyle, and quality of life [44].

Compared with consumers high in health consciousness, consumers low in health consciousness have less motivation to engage in healthy actions [43, 44], choose unhealthy foods [43–45]. Forthofer and Bryant [46] opined that consumers high in health consciousness are considered as “targets of greatest opportunity” (p. 37) since such consumers are more expected to engage in healthy actions, are considered to buy organic food that includes higher nutritional values because of health advantages involved [43–45]. Healthy conscious consumers used to express interest in matters concerning food to avoid any food that is dangerous to their health and wellbeing [44]. Thus, when making purchase intention, healthiness is a significant determinant and the parameters for these consumers [47]. Earlier empirical studies reported that a consumer’s health consciousness is significantly and positively linked with his/her purchase intention toward organic foods [44, 48, 49]. Therefore, the following hypothesis is advanced:

- Hypothesis 1: Health consciousness has a positive and significant effect on the purchase intention.

### Environmental concern

Environmental concern has received a lot of attention in academia and business because of the increase in air pollution due to carbon dioxide emissions [50]. Environmental behaviorists always treat the environmental concern construct as an individual consumer’s degree of concern related to environmental matters [51]. Lee [52] defined environmental concern as consumers’ assessment of environmental factors deciding to buy a particular product or service and reported environmental concern influences consumers’ purchase intention. Therefore, the environmental concern construct is vital in understanding consumers’ purchase intention of organic product that is friendly to the environment. Even though consumers’ environmental concern is new inclinations that can be influenced by other factors [53]. Kaygusuz [50] reported that consumers’ environmental concern is linked with such factors as knowledge, education level, and experiences. For example, consumers’ positive feelings acquired through experiences in his/her natural environment can motivate environmental concerns [23, 53]. Similarly, the state of the natural surroundings can affect consumers’ environmental concerns. A typical example of this is China’s air pollution. Consumers are said to be high in environmental concern if they identify the impact of their activities or actions on the natural environment [53].

In the context of food consumption, more consumers are nowadays vegetarians compared to a few years back [54]. Consumers are not only adopting a vegetarian lifestyle because of health concerns; consumers now accept a vegetarian lifestyle because of the environment [50]. Organic food products, which are produced using natural farming techniques, decrease the contamination of groundwater and soil contamination because pesticides and fertilizers that are destructive to the environment are not applied to the soil. Earlier studies reported that organic product consumers buy organic food by considering environmental issues [54]. Prior investigations submitted that environmental concern is directly and significantly influences consumers’ attitude towards organic products [23, 53] and consequently influences their purchase intention [51]. The idea is that high environmental concern consumers always have a positive attitude toward organic food which increases their level of purchase intention. Therefore, the following hypothesis is advanced:

- Hypothesis 2: Environmental concern has a significant positive effect on the purchase intention.

### Consumer innovativeness

Innovativeness is a consumer innate which refers to the “predisposition to buy new and differentiated products and brands rather than remain with previous choices and consumer patterns” [55]. Innovativeness can also be seen as individuals’ predisposition to willingly accept change and/or attempt new products or services rather than taking on the general unhealthy consumption patterns [56, 57] and willingly buy new products/services more quickly and frequently than others [57]. Therefore, consumer innovativeness refers to the consumers’ desire to look out for novelty and arousal from particular new products/services [57]. Midgley and Dowling [58] conceptualized consumer innovativeness as the extent to which consumers are receptive to new products/services, ideas, and choose to accept new technology, not considering the other consumers’ experiences. Venkatraman, [59] categorizes innovativeness in two: sensory and cognitive innovativeness. Cognitive innovativeness is the consumers’ predisposition to rationalize, reflect, and solve problems. In this case, the consumers are always looking for a novel experience that may arouse their mental actions or activities. Moreover, it is fruitful to note that manufacturers should increase their entrepreneurial skills and acquisition [60] to provide health-conscious customers with better health services.

Healthy conscious consumers concurrently consider product assimilation and differentiation while intending to purchase a particular product or service [54]. The tendency to accept unconventional lifestyles is what differs from health-conscious consumers to conservative consumers [57, 60]. Health-conscious consumers are generally willing to try a new product such as organic food can be described as “global consumer innovativeness” [57, 60]. Consumer innovativeness is likely to be determined by novelty-seeking, need for uniqueness, and stimulation needs [57]. Earlier studies have established consumer innovativeness construct as a significant factor affecting consumers’ product adoption especially organic food [57, 60]. Thus, innovative consumers are more likely and easily accept new products/services and/or technologies [57]. Besides, creative consumers of organic food generate higher attitudes related to organic food attributes than conservative consumers [56]. Given that, consumer innovativeness level, which is regarded as a consumer’s characteristic [58], could positively affect the linkage between consumers’ product innovativeness and organic food purchase intention in this study. Therefore, the following hypothesis is advanced:

- Hypothesis 3: Consumer innovativeness has a significant positive effect on the purchase intention.

### Healthy lifestyle

A healthy lifestyle refers to the customers’ modifications in health behavior following the constant purchase of organic food [61]. In this study, a healthy lifestyle refers to behaviors that health-conscious customers consider and accept which sustain their well-being such as constant intake of organic products [62]. For instance, a healthy lifestyle includes controlling weight and regular eating of organic fruits or organic vegetables [61, 62]. A healthy consumption lifestyle is consumer orientation towards preventing product that causes health problems [63]. Therefore, a healthy consumption lifestyle is the customers’ consumption activities and actions, including consumption of organic food for better health and life prosperity [64]. Healthy conscious consumers are more likely to make efforts and activities that are beneficial for their health, reduce body weight by doing sports activities, and consume organic food to enhance their healthy lifestyle [64]. Since they have a positive attitude and intentions toward purchasing organic food products. Therefore, organic food products are important for consumers’ healthy lifestyles.

Customers who consider the health benefit of a product, taste, and environmental protection and consider improving their lifestyle are the potential customers and consumers of organic food [15, 61]. Customers from the healthy lifestyle category are orientated toward health and are pleased with the lifestyle that concentrates on health [15]. In consumer behavior literature, scholars’ reported that consumers’ lifestyle will decide their consumption attitude and behavior towards purchasing organic food [15, 65]. In their study, scholars [65] advocated that a healthy lifestyle influences a healthy conscious customer’s behavioral intention towards organic and environmentally friendly products. Their findings are in line with the finding of Sagheb, Ghasemi, and Nourbakhsh [66] and Güney, and Giraldo [64] reported that a healthy lifestyle is positively related to a health-conscious consumer’s attitude towards the organic product. Hence, the higher the health-conscious consumers’ propensity to eat organic food that will improve their healthy lifestyle, the increased the consumers’ intention to purchase organic products. Therefore, the following hypothesis is advanced:

- Hypothesis 4: Purchase intention has a significant positive effect on the healthy lifestyle.

### Brand credibility identification

The concept of credibility is generally distinguished as the trustworthiness of an entity’s (e.g., brand) intents at a specific instance [67]. Scholars have conceived credibility mainly based on two dimensions: [1] trustworthiness, and [2] expertise [68]. Ergo, brand credibility has been described as the believability of the product information enclosed in a brand, which necessitates that consumers recognize that the brand has the expertise (e.g., ability) and trustworthiness (e.g., intents) to constantly offer what has been pledged [68, 69]. Thereby, consumers identify the brand’s credibility by engaging in an appraisal mechanism that outlines the cumulative credibility of a brand [70]. According to the brand signaling theory, consumers identify the credibility based on the underlay produced by the brand’s echoed expertise and trustworthiness. This phenomenon involves the aggregated influences attached to the brand’s past and present communicated commitments and marketing strategies that symbolize credibility to the consumers [71]. The consumers evaluate the informational content of the brand to evaluate or identify its credibility. On the other hand, a plethora of literature suggests that brand function as an indicator has clarified that brand credibility can be eroded if a brand does not provide what has been promised [71, 72]. The trust determination theory also sheds light on the significance of the credible source of information, it emphasizes that information from a trustworthy source can formulate favorable behavior [73]. Since the information impending from the brand appraised as credible by the consumer can diminish the suspicions and act favorably.

Most consumer behavior research also affirmed that brand credibility identification is a prevailing psychological phenomenon that prompts the purchasing instinct of consumers [72]. For instance, studies have explored the brand credibility positively predicts behavioral outcomes such as consumer satisfaction [71, 72] and purchase-making decisions [74]. However, from the risk communication perspective, owing to health safety potential apprehensions consumers’ take more conscious decisions about the selection of innovative food products [71, 73]. Psychological theories such as the heuristic-systematic enlightened that individuals are influenced by the communicated product information and contemplate the source of information (e.g., brand credibility) [71]. The information processing mechanism, thus, includes the evaluation of the brand credibility that serves as a functional cue to reduce apparent uncertainties. Lassoued and Hobbs [74] described that food consumers sense more confidence about brands with a good reputation for food safety. Within the food consumption context, the literature suggests that brand credibility has a substantial influence on consumers’ buying behavior [67], consumer trust in the usage of innovative foods [75], and reduction in food safety concerns [76]. Therefore, a higher level of consumer perceived identification of brand credibility generates more inclination towards purchasing the healthy foods’ product and we hypothesized that:

- Hypothesis 5: Brand credibility identification has a significant positive effect on the healthy foods’ purchase intention.

### Brand image identification

Brand image is an aggregate term representing the consumers’ overall perceptions, feelings, uniqueness of associations, favorability, beliefs, appraisals about a brand developed through their experiences [77, 78]. The consumer identifies brand image by inferring the association, attitude, belief, evaluation, and overall impression of a brand [79]. Therefore, brand image is associated with consumers’ understanding of the brand that develops over time and characterizes the symbolic meaning of consumption [80]. Alamsyah, Othman, and Mohammed [81] noted that brand image reflects a unique identification to consumers from other counterparts’ brands in the marketplace. Hence, a favorable brand image is indispensable for the brands concerning consumer behavior [82]. In this regard, it plays a significant role in assisting consumers in making purchasing decisions whether to purchase or repurchase a particular [81]. Therefore, brand image is an all-inclusive demonstration of numerous aspects of the brand in the minds of consumers [83]. According to Hien, Phuong, Tran & Thang [55] the consumer responses to a brand’s marketing practices are constantly determined by an affirmative association between a brand and its anticipated characteristics symbolic meanings in the minds of consumers.

In verily, setting up a good brand image identification is an imperative undertaking of food marketers and enterprises [82]. Consumers when encounter marketing activity of a brand with a prior positive image, it is utmost expected they will undergo to purchase [84]. In the context of food marketing, ample research has been carried out that affirms the brand image drives the consumers’ inclination towards purchasing organic food [85] genetically modified food [86, 87], and bio-fortified food [81, 88]. The literature suggested that a favorable brand image helps food products gain a competitive advantage and purchase intention [55, 80]. Similarly, another recent study on food marketing noted that brand image is established to influence consumer food purchase intention [81] positively. Several studies on healthy food consumers’ behavior suggest that consumers are motivated to purchase once they identify the brand’s image based on its established traits and characteristics [85, 87]. Hence, greater brand image identification will positively influence the consumers’ intention to purchase healthy food and it is hypothesized that:

- Hypothesis 6: Brand image identification has a significant positive effect on the healthy foods’ purchase intention.

## Method and materials

### Research settings: health online community

Facebook was launched in October 2003 by Mark Zuckerberg. In February 2010, 400 million users registered with Facebook. Then, community pages were announced on Facebook in April 2010. Currently, Facebook has 2.85 billion monthly active users [89]. Facebook allows us to share text and visual content about users’ daily lifestyles including health and fitness. The healthy foods and healthy lifestyle community on Facebook are chosen as the focus of this study. The data were collected on social media Facebook to test the research model.

The study was granted ethical approval by the Research Ethics Committee of the Universiti Putra Malaysia and signed informed consent was obtained from the subjects 18+ years of age for data to be used for research purposes. Furthermore, all methods were carried out in accordance with relevant guidelines and regulations.

### Measurement and validity

Based on the literature review, the design of questionnaire items was stemmed. The seven variables were measured using Likert scales, with a total of 42 items. All items used five-point Likert scales. The details about all items can be found in Table 1. To augment the content validity of this questionnaire, 30 experts were contacted to participate in this study and only 10 experts were responded and validated this questionnaire. Then, we revised the questionnaire based on experts’ feedbacks. Finally, the pilot study took place before actual data collection.

**Table 1:** Items by Construct

Constructs	Number of Items	Sources
Healthy foods’ purchase intention	6	Shin & Severt (2020); Yazdanpanah, & Forouzani (2015)
Health consciousness	8	Ali, et al. (2020); Smith & Paladino (2010)
Environmental concern	6	Lee (2008)
Consumer innovativeness	6	Zhang, et al. (2020)
Brand credibility identification	5	Spry et al. (2009)
Brand image identification	5	Cretu & Brodie (2009)
Healthy lifestyle	6	Güney & Giraldo (2019)

### Data collection procedure

Based on the locus of the study employed a cross-sectional online survey for the data collection from the online health community users. The online survey was conducted for a period of three months from January to March 2021. For that reason, hired enumerators posted the link to the questionnaire on their own Facebook accounts to the selected online healthy communities such as ‘Diet Suku Suku Separuh’ (469,000 followers), ‘Hiking, and Camping around Malaysia’ (351,200 followers), and ‘Healthy Malaysia’ (332 followers). The enumerators also engaged with the online community by liking posts and following health accounts. A total of 241 questionnaires were received and validated for analysis.

## Results

### Descriptive statistic

As shown in Table 2, the sample descriptive statistics, there were largely more females (66.6%) than males (33.3%). Approximately 41.5% of the respondents were between 19 to 29 years. Regarding nationality, the majority was Malaysian (83.8%), while international respondents consisted of 16.2%. Nearly 45.6% were Malay, followed by Chinese (21.2%) and Indian (17%). There were predominantly single (51.5%) regarding the marital status. Concerning educational qualifications, approximately 42.3% of the respondents had Bachelor’s degree. Finally, the respondents’ income reported that the majority had below RM3000 (35.3%).

**Table 2:** **Sample Descriptive Characteristics (*N*** **= 241)**

Profile		Frequency	Percentage
**Gender**			
Male		81	33.6
Female		160	66.4
**Nationality**			
Malaysian		202	83.8
International		39	16.2
**Race**			
Malay		110	45.6
Chinese		51	21.2
Indian		41	17.0
Others		39	12.1
**Age**			
19–29		100	41.5
30–39		68	28.2
40–49		48	19.9
50–59		21	8.7
Above 60		2	0.8
Below 18		2	0.8
**Marital status**			
Single		124	51.5
Married		107	44.4
Divorce		4	1.7
In a Relationship		2	0.8
Widow		4	1.7
**Educational qualifications**			
PhD		18	7.5
Master’s Degree		40	16.6
MBA		11	4.6
Bachelor’s degree		102	42.3
Certificate or diploma		65	27.0
Senior High School		5	2.1
**Income**			
Below RM3,000		85	35.3
RM3001 - RM6,000		72	29.9
RM6,001 - RM9,000		32	13.3
RM9,001 - RM12,000		12	5.0
RM12,000 - RM15,000		5	2.1
RM15,001 and above		6	2.5
None		29	12.0

### Measurement model: individual items reliability and internal consistency reliability

**Fig. 3:**
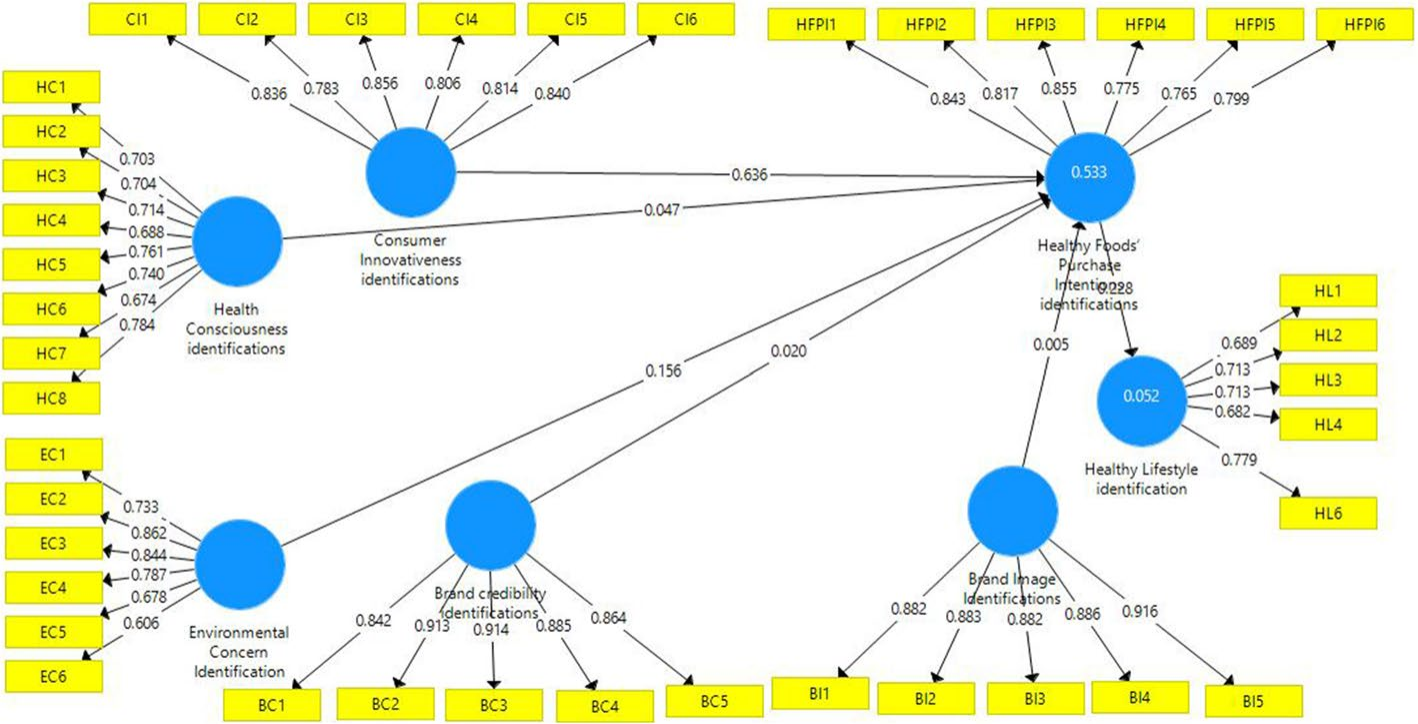
Measurement Model

In this study, item reliability was assessed using factor loadings (refer to Table 3). In this study, all items loaded above 0.7, only a few items reported loadings below above 0.6 which is acceptable (refer to Table 3 and Fig. 3). The internal consistency reliability was measured using composite reliability [90]. Table 3 reported that all the constructs exhibit adequate internal consistency above 0.7 as requirements (Hair et al., 2013).

**Table 3:** Factor Loadings, Composite Reliability, and Average Variance Extracted

Constructs	Items	Loadings	CR	AVE
Brand credibility identification	BC1	0.842	0.947	0.782
	BC2	0.913		
	BC3	0.914		
	BC4	0.885		
	BC5	0.864		
Brand image identification	BI1	0.882	0.950	0.792
	BI2	0.883		
	BI3	0.882		
	BI4	0.886		
	BI5	0.916		
Consumer innovativeness	CI1	0.836	0.926	0.677
	CI2	0.783		
	CI3	0.856		
	CI4	0.806		
	CI5	0.814		
	CI6	0.840		
Environmental concern	EC1	0.733	0.888	0.573
	EC2	0.862		
	EC3	0.844		
	EC4	0.787		
	EC5	0.678		
	EC6	0.606		
Health consciousness	HC1	0.703	0.897	0.521
	HC2	0.704		
	HC3	0.714		
	HC4	0.688		
	HC5	0.761		
	HC6	0.740		
	HC7	0.674		
	HC8	0.784		
Healthy foods’ purchase intention	HFPI1	0.843	0.919	0.656
	HFPI2	0.817		
	HFPI3	0.855		
	HFPI4	0.775		
	HFPI5	0.765		
	HFPI6	0.799		
Healthy lifestyle	HL1	0.689	0.840	0.513
	HL2	0.713		
	HL3	0.713		
	HL4	0.682		
	HL6	0.779		

Note: AVE = Average variance extracted CR = Composite reliability

With regards to the convergent validity, convergent validity in this study was determined using composite reliability (CR), and average variance extracted (AVE). As shown in Table 3, CR is higher than 0.7 and AVE is higher than 0.5 [91] (refer to Table 2 and Fig. 3).

### Discriminant validity

In this study, discriminant validity was measured using the HTMT ratio as this method was reported as the reliable method for measuring discriminant validity [92]. The HTMT ratio reported that discriminant validity was satisfied in this study. The HTMT values were within the yardstick of 0.85 [92] (refer to Table 4).

**Table 4:** Discriminant Validity Heterotrait-Monotrait Ratio (HTMT)

Constructs	1	2	3	4	5	6
1. Brand image identification						
2 Brand credibility identification	0.827					
3 Consumer innovativeness	0.329	0.482				
4 Environmental concern	0.328	0.382	0.292			
5 Health consciousness	0.371	0.425	0.443	0.611		
6 Healthy foods’ purchase intention	0.296	0.415	0.765	0.412	0.447	
7 Healthy lifestyle	0.343	0.382	0.315	0.503	0.698	0.258

### Analysis of the structural model

**Fig. 4:**
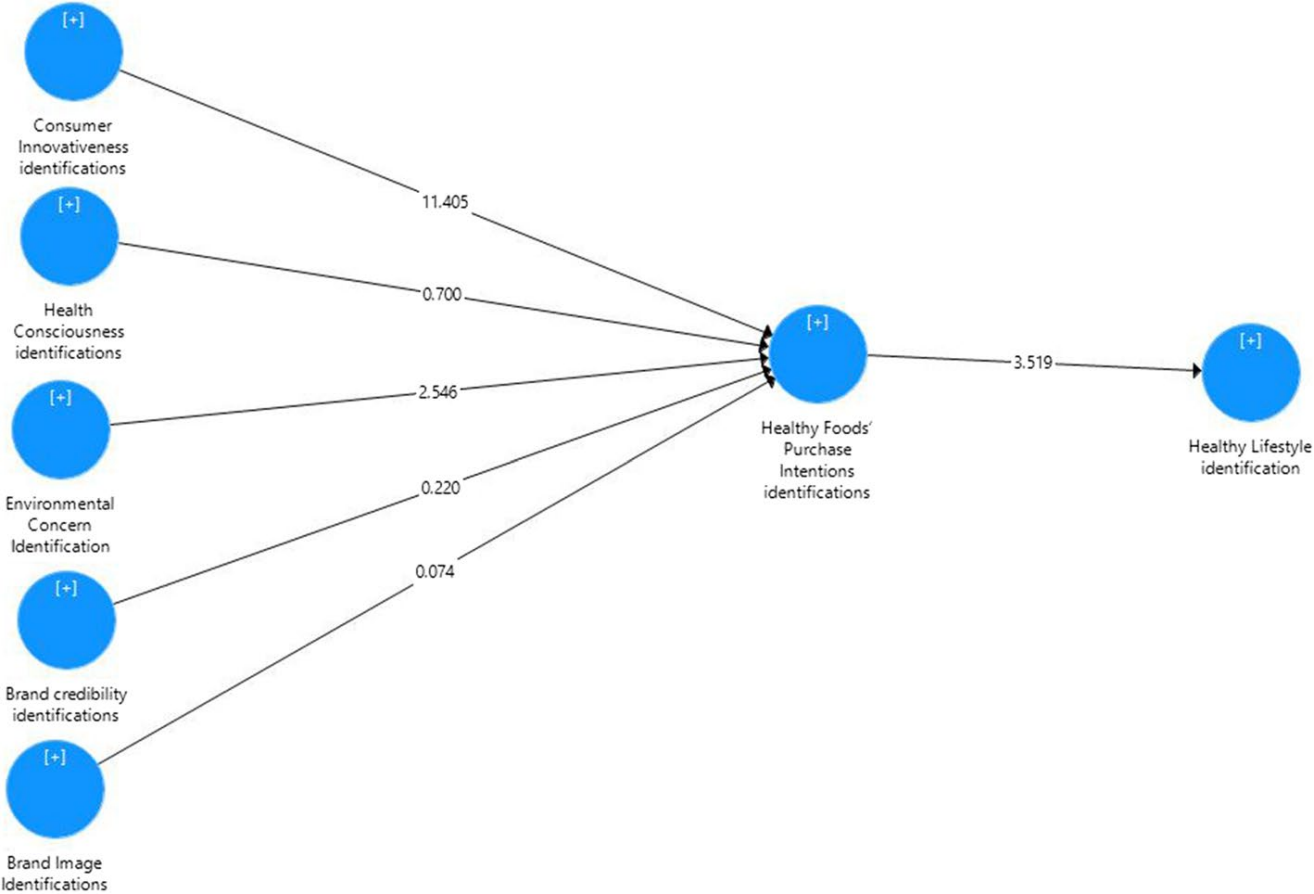
Structural Model Main Effect

To test the path coefficients’ significance, a bootstrapping was employed via 5000 subsamples which provide t-values and *p* values of the parameters [93]. Regarding the R2 values, the study model explains 53.3% of the healthy foods’ purchase intention variance. Therefore, brand credibility identification, brand image identification, consumer innovativeness, environmental concern, and health consciousness were significant contributors to the prediction of foods’ purchase intention in this study (Refer to Fig. 3). Also, the study model explains 5.2% of healthy lifestyle which is reported by the R2 values (Refer to Fig. 4). Therefore, purchase intention explains 5.2% on healthy lifestyle.

The study model predictive relevance (Q^2^) [94] is greater than zero indicate adequate model predictive relevance [94]. The findings of this study confirmed that the Q^2^ value for the dependent variables is acceptable (healthy foods’ purchase intention = 0.319) and (healthy lifestyle = 0.020). Another criterion to assess the structural model is the effect size (f^2^). Cohen (1988) classified f^2^ of 0.02, 0.15, and 0.35 as small, medium, and large respectively. The f^2^ shown in this study is an acceptable range mainly large and small based on Cohen’s (1988) classification (refer to Table 5).

**Table 5:** Structural Model Assessment Direct Effect

Relationships	Beta Values	Standard Deviation	T Statistic	P Values	Decision
Brand image identification - > Healthy foods’ purchase intention	0.005	0.071	0.074	0.941	Not Support
Brand credibility identification - > Healthy foods’ purchase intention	0.020	0.091	0.220	0.826	Not Support
Consumer innovativeness - > Healthy foods’ purchase intention	0.636	0.056	11.405	0.000**	Supported
Environmental concern - > Healthy foods’ purchase intention	0.156	0.061	2.546	0.011*	Supported
Health consciousness - > Healthy foods’ purchase intention	0.047	0.067	0.700	0.484	Not Support
Healthy foods’ purchase intention - > Healthy lifestyle	0.228	0.065	3.519	0.000**	Supported

The findings indicated no positive relationship between brand image identifications and healthy foods’ purchase intention (β = 0.005; t = 0.074; *p* = 0.941). The findings indicated a positive relationship between consumer innovativeness and healthy foods’ purchase intention (β = 0.636; t = 11.405; *p* = 0.000). The findings indicated a positive relationship between consumer innovativeness and healthy foods’ purchase intention (β = 0.636; t = 11.405; *p* = 0.000). This finding shows that the respondents who rated consumer innovativeness higher also indicated higher scores on the healthy foods’ purchase intention scale. (Refer to Table 6). The findings indicated a positive relationship between environmental concern and healthy foods’ purchase intention (β = 0.156; t = 2.546; *p* = 0.011). This finding shows that the respondents who rated environmental concern higher also indicated higher scores on the healthy foods’ purchase intention scale. (Refer to Table 6). The findings indicated no positive relationship between health consciousness and healthy foods’ purchase intention (β = 0.047; t = 0.700; *p* = 0.484) (Refer to Table 5).

**Table 6:** Structural Model Assessment Indirect (Mediating) Effect

	Beta Values	Standard Deviation	T Statistics	P Values	97.5%	97.5%	Decision
Brand image identifications - > Healthy foods’ purchase intention - > Healthy lifestyle	0.001	0.019	0.064	0.949	0.042	0.040	Not Supported
Brand credibility identifications - > Healthy foods’ purchase intention - > Healthy lifestyle	0.005	0.024	0.195	0.846	0.050	0.055	Not Supported
Consumer innovativeness - > Healthy foods’ purchase intention - > Healthy lifestyle	0.145	0.040	3.676	0.000**	0.235	0.213	Supported
Environmental concern - > Healthy foods’ purchase intention - > Healthy lifestyle	0.036	0.021	1.660	0.097	0.091	0.082	Not Supported
Health consciousness - > Healthy foods’ purchase intention - > Healthy lifestyle	0.011	0.019	0.560	0.575	0.061	0.052	Not Supported

Note: **Significant at 0.01 (1-tailed), *Significant at 0.05 (1-tailed)

The findings indicated a positive relationship between healthy foods’ purchase intention and healthy lifestyle (β = 0.228; t = 3.519; p = 0.000). This finding shows that the respondents who rated healthy foods’ purchase intention also indicated higher scores on the healthy lifestyle scale. Finally, healthy foods’ purchase intention was found to mediate the relationship between consumer innovation and healthy lifestyle (β = 0.145, t = 3.676, *p* = 000). Surprisingly, no mediation effect of healthy foods’ purchase intention was found in this study on the relationship between brand credibility identification, brand image identification, environmental concern, and health consciousness on healthy lifestyle (Refer to Table 7).

**Table 7:** Effect Size

Constructs	Healthy foods’ purchase intention	Healthy lifestyle
Brand image identification	0.000	
Brand credibility identification	0.000	
Consumer innovativeness	0.629	
Environmental concern	0.037	
Health consciousness	0.003	
Healthy foods’ purchase intention		0.055

## Discussion

Prior literature has contributed to developing an understanding of organic and healthy food consumption. These studies underpinned several antecedents of such as health consciousness [21] brand trust [95] and environmental concerns [51] that predict the purchasing intention towards organic food [13] nutrients food [14]. However, interestingly most of these studies only researched whether the social factors [23, 40] or individual psychological traits [25, 44], or branding factors [71] in determining healthy food consumption. These studies endure a void in the literature and to our knowledge, no prior studies integrated these facets to understand the phenomenon of healthier food consumption. These studies theoretically argue that multiple factors equally influence consumers. For example, according to trust determination and brand signaling theory when a consumer encounter coming information from a brand, the consumer evaluates the information based on the prior acquired level of trust about the brand [73]. At the same time, the theory of planned behavior maintains that such individual positive predispositions influence their purchase intentions [40, 96]. Similarly, the individual traits notion suggests that individual traits such as environmental concerns influence one’s behavior.

Furthermore, prior literature indicated that individual decisions about purchasing are not only influenced by the socio-cultural and individual abilities to carry out behavior in question [71]. The information processing phenomenon is thus complex and people also look into the other attributes of the product [97]. For instance, organic food can serve as an attribute of a healthier food product, which impacts individuals’ decision-making process. According to the signaling theory people usually make choices based on accessible information resource. Plentiful literature suggests that if people are aware of the health benefits of organic food owing to the product labeling reflecting product attributes [22], this freely accessible information can improve their adoption level [98]. However, the level of adoption or purchase would depend of certain perceptions like information source credibility [97]. Therefore, consumers get a signal to act upon based on the quality and a source of information about the products provided to them. These signals aid consumers take decisions about the adoption of the products in question. On the other hand, TPB explained the social and individual factors such as subjective norms, behavioral control and attitude as determinants of the individual’s choice. This study argues that apart from the social factors delineated in TPB, there is critical role of accessible information in determining one’s behavioral outcomes. As mentioned earlier, fewer studies have been conducted to understand the combined effect of this crucial factor [44]. By integrating TPB and signaling theory, this study examines the role of the TPB and brand signaling mechanisms in the purchase intention of healthier food. Drawing on the analogy of previous research, the current study integrated these perspectives in a single study to understand the phenomenon of healthy lifestyle adoption.

This study is among the first to address such gaps in the literature by combining the three TPB components with signal quality to predict intention. in doing so study has re-conceptualized the role of the TPB elements and advanced the model by adding perspective from the signaling theory. The information source credibility and image identification serve as antecedents that enable consumers to take a decision about the food product [22]. The research identified that virtuous signal should be noticeable (e.g., brand image) and credible (e.g., brand credibility). In comparison, the consumer innovativeness, health and environmental consciousness are likely to determine the overall healthier food consumption pattern. The study theoretically underscored the interplay between TPB factors and brand signaling factor of information resources. Theoretically, once people encounter any information, they start psychological, cognitive and affective processing of received information. According to the signaling and TPB, they start deriving and interpreting that information by utilizing their psychological and social resources. Based on the signaling theory the present study postulated that organic food intention would be reliant on the properties of the signal [1] clarity (e.g., brand image identification) and [2] credibility (e.g., brand credibility identification) [99]. For instance, when Malaysian consumers perceived and identified the organic food advocating brand credible and known with an established image, there would be more adoption. This is consistent with the theoretical assumptions of signaling theory that suggests clarity and credibility leads towards fewer or lack of ambiguity in the information conveyed. This consistency between consumers’ past perception about the brand credibility provokes their self-confidence in the assertions made about the product attributes.

Although organic food is related to healthier food patterns, in a country like Malaysia, whereby compared to Europe or the USA, organic food consumption is less popular. Similarly, organic products’ functional attributes to help in reduction of obesity is also less known to the people. This present study integrated individual factors such as consumer innovativeness and attitude-related factors health and environmental consciousness to get insightful evidence in the context of Malaysia. The study argues that consumer innovativeness and consciousness of the Malaysian individuals’ interplay with information resources. To a theoretically convinced point, a brand promoting organic food as a healthier lifestyle (e.g., counter obesity) sends signals to likely consumers. However, receivers of such signals will employ their social and psychological resources while making the decision about the organic food adoption. The credible and known brand can convey a signal that can enable consumers to rapidly comprehend and delineate the functional attributes of organic food; however, less extent of consumer innovativeness and consciousness can prevent such inclination.

Thus, the study fills a theoretical knowledge gap in the Malaysian context and examined several hypotheses using PLS (SEM) approach. The outcomes of H1 revealed interesting information that health consciousness not significantly predicts the purchase intention of healthy food. These results are, although not consistent with prior studies that recommended health consciousness as a potential antecedent of purchase intention of healthy food [25, 48, 49]. However, prior terror management theory (TMT) noted that “fear of death haunts the human mind”; thereby, individuals’ extent of consciousness is related to the degrees of threat they feel [100]. The results are not surprising in the context of the TMT standpoint because the underpinning question of this research is about a generally healthy lifestyle. Furthermore, information-seeking theories also justify these results. For instance, findings of studies on serious diseases reported entirely different aspects. People are found more concerned about their health and seek more information when they feel more threatened (i.e., cancer), this, in turn, improves their level of health consciousness [20, 23]. Furthermore, people are exposed to a lot of marketing activities by other different food brands advocating their safety and nutritional value. Hence people may have perceived other foods are also safe and pose fewer threats to their health. In contrast, findings of the influence of the environmental concerns posited in H2 were supported and found consistent with prior studies [54]. This suggests that people perceive organic food as more sustainable consumption and production phenomenon. In a similar vein, the most interesting results have been revealed about the influence of consumer innovativeness characteristics that positively influence purchase intention. Scholars [56, 57] referred to one’s innovativeness characteristics as individual traits. The persons who uphold these traits are always inclined towards the adoption of novel and innovative products. To this point, the hypothesized proposition of this study has been verified and can be concluded that the sample has perceived the organic products as more novel than the accessible prior products and hence, supported the favorable purchasing behavior [72].

Despite the growing recognition of the significance of brand credibility and image in marketing literature, little effort has been made to investigate how consumers perceived brand credibility and image influence food consumption patterns. The past literature applying theories such as brand signaling theory [69, 71] and trust determination [73] recommends that brand credibility and brand image identifications of products are critical to comprehend consumer behavior [77]. This study employed a holistic background that investigates the influence of these branding elements on the purchase intention of organic food brands. However, PLS (SEM) findings do not support the postulations proposed. Apparently, these findings are in contradictions with the prior studies and branding theories such as brand signaling theory that advocates the associations of brand credibility and image with consumers’ intention [72]. Moreover, most of the past research identifying the strong relationship between brand credibility and image has been carried out in the western context [70]. Since the context of healthy food (i.e., organic) is quite novel in Asian markets and brands have to still undergo marketing practices to develop brand awareness. However, H6 has supported the direct influence of purchase intention of healthy food on the adoption of a healthy lifestyle. This was quite obvious as several factors contribute to the formulation of purchasing behavior**.**

Lastly, considering very limited marketing practices of organic food brands in Asian markets the mediating influence of healthy foods’ purchase intention was not identified as a mediating variable among the relationship between brand credibility identification, brand image identification, environmental concern, and health consciousness. Only, mediation of healthy foods’ purchase intention was found significant between consumer innovativeness and healthy lifestyle. Although this is quite surprising seemingly, there are very few brands available in the market and indeed consumers are less engaged in buying healthy food. Interestingly, those consumers with a higher degree of innovativeness are inclined to do so, in contrast, results depict those consumers are uncertain about the benefits of healthy food usage. This is consistent with theories that imply consumers’ brand awareness and contextual factors play their part in developing consumer behavior [95].

### Managerial implications

Besides, conceptually the health consciousness includes extensive facades such as individual lifestyles, interests, and interpretations about own health [19, 21]. Still, these results have the important managerial implication that people are not well conscious about the benefits of using organic food. In the case of Asian markets, there are very few companies operating and providing information about the usage of healthy food. Most of the information available through less credible sources such as online platforms. This, in turn, increased the uncertainties among the people, the results of current studies suggest that brands may adopt crisis marketing strategies while prompting healthy lifestyle products. On the other hand, government and non-government organizations also may make people more conscious using several means of communication. For example, results suggest that people are not health conscious and reluctant in adopting healthy food patterns. To illustrate more, Malaysia is ranked among the nations that have a higher ratio of diabetes due to obesity among people. Therefore, the promotion of healthier food is becoming a public health issue and must be administrated by promoting more awareness. Likewise, obesity, other issues such as iron, vitamin, and mineral deficiencies are linked with unhealthy food consumption and quite common in the Asian region. The psychological terror management model suggests that when people realize the scarcity they adopt new habits [100]. Thus, it is recommended that organizations must aware of the people about pros of adopting healthier lifestyles. Furthermore, the promotional campaigns also highlight the growing deficiencies drawbacks to one’s health so that people adopt a healthier lifestyle.

## Conclusion

The motivation of the study is due to the high number currently obsessed or overweight in Asia. Specifically, Malaysia makes it necessary for the researchers to understand the antecedents and outcomes of health-conscious consumers’ purchase intention of organic food products. As an important area in green product research, organic food purchase intention of consumers have received more research attention. This paper is set to develop and propose a conceptual framework for healthy organic foods’ purchase intention. This paper is planned as a guide for future studies to use and validated as a foundation for quantitative studies to investigate the health-conscious consumers’ purchase intention of organic food products. Drawing on TPB, the antecedents and outcomes of health-conscious consumers’ purchase intention of organic food products healthy lifestyle among the citizens of Asian countries were reviewed and research hypotheses were offered. Finally, recommendations will be offered to various stakeholders on how to improve a healthy lifestyle in Asia. This conceptual analysis applied TPB as a basis to develop the antecedents and outcomes of consumers’ purchase intention of organic food products among the citizens of Asian countries. This conceptual model proposed that health-conscious consumers’ health consciousness, environmental concern, and innovativeness will predict health-conscious consumers’ food purchase intention and consequently improve their healthy lifestyle. Future research will have to consider encouraging corporate engagement with communities [101] may influence the communities’ healthy lifestyle. Beyond the brand image of health products, improving healthcare companies’ favorable corporate reputation [82, 102] may lead to a higher degree of healthy organic foods’ purchase intention theoretically. Indeed, this study, if validated, will offer empirical evidence that could help policy-makers and Malaysian citizens increase the use and purchase of organic food products, which will certainly improve their healthy lifestyles. Moreover, internalizing cultural diversity [103, 104] and mental health [105] among Malaysian people can be a central focus of future study in the healthcare industry in Asia.

## Data Availability

The data that support the findings of this study are available from the corresponding author upon reasonable request due to ethical and privacy restrictions.

## References

[1] Chiang J, Arons A, Pomeranz JL, Siddiqi A, Hamad R. Geographic and Longitudinal Trends in Media Framing of Obesity in the United States. Obesity. 2020;28(7). 10.1002/oby.22845 PMC7311269 32475076

[2] WHO. Obesity and overweight [Internet]. 2020. Available from: https://www.who.int/news-room/fact-sheets/detail/obesity-and-overweight

[3] Rachmi CN, Agho KE, Li M, Baur LA. Stunting, Underweight and Overweight in Children Aged 2.0–4.9 Years in Indonesia: Prevalence Trends and Associated Risk Factors. Zhang Y, editor. PLoS One [Internet]. 2016 May 11;11(5):e0154756. Available from: https://doi.org/dx.plos.org/10.1371/journal.pone.0154756 10.1371/journal.pone.0154756 PMC4864317 27167973

[4] Biswas T, Magalhaes RJS, Townsend N, Das SK, Mamun A. Double Burden of Underweight and Overweight among Women in South and Southeast Asia: A Systematic Review and Meta-analysis. Vol. 11, Advances in Nutrition. 2020. 10.1093/advances/nmz078 PMC7442413 31634389

[5] Lubis SM, Batubara J, Damanik HA, Fattah M. Association of fat mass and obesity-associated gene rs9939609 variant with early onset obesity among bataknese and Chinese children in Indonesia. Int J Pediatr Endocrinol [Internet]. 2015 Dec 28;2015(S1):P73. Available from: https://ijpeonline.biomedcentral.com/articles/10.1186/1687-9856-2015-S1-P73

[6] Chamie J. World Population: 2020 Overview | YaleGlobal Online. YaleGlobal Online. 2020.

[7] Tee JYH, Gan WY, Lim PY. Comparisons of body mass index, waist circumference, waist-to-height ratio and a body shape index (ABSI) in predicting high blood pressure among Malaysian adolescents: A cross-sectional study. BMJ Open [Internet]. 2020 Jan 12;10(1):e032874. Available from: https://bmjopen.bmj.com/lookup/doi/10.1136/bmjopen-2019-032874 10.1136/bmjopen-2019-032874 PMC7044891 31932391

[8] Lee YY, Tan D, Siri J, Newell B, Gong Y, Proust K, et al. The role of public health dietary messages and guidelines in tackling overweight and obesity issues [Internet]. Vol. 26, Malaysian Journal of Nutrition. 2020. p. 31–50. Available from: http://nutriweb.org.my/mjn/publication/26-1/Vol26(1) 3.mjn.2019.0084 Yi Yi Lee (online first) updated.pdf

[9] Goh E Von, Azam-Ali S, McCullough F, Roy Mitra S. The nutrition transition in Malaysia; Key drivers and recommendations for improved health outcomes. BMC Nutr. 2020;6(1). 10.1186/s40795-020-00348-5 PMC7322903 32612845

[10] Yan J, Zheng Y, Bao J, Lu C, Jiang Y, Yang Z (2020). How to improve new product performance through customer relationship management and product development management: evidence from China. J Bus Ind Mark.

[11] Wenzel H, Hauschild M, Alting L, Overcash M. Environmental assessment of products volume 1: Methodology, tools, and case studies in product. Int J Life Cycle Assess [Internet]. 1999 Jan;4(1):6–6. Available from: http://link.springer.com/10.1007/BF02979388

[12] Mosier SL, Thilmany D. Diffusion of food policy in the U.S.: The case of organic certification. Food Policy [Internet]. 2016 May 1 [cited 2021 May 21];61:80–91. Available from: https://linkinghub.elsevier.com/retrieve/pii/S0306919216300033

[13] Seufert V, Ramankutty N, Mayerhofer T. What is this thing called organic? – How organic farming is codified in regulations. Food Policy [Internet]. 2017 Apr;68:10–20. Available from: https://linkinghub.elsevier.com/retrieve/pii/S0306919216300690

[14] Taghikhah F, Voinov A, Shukla N, Filatova T. Exploring consumer behavior and policy options in organic food adoption: Insights from the Australian wine sector. Environ Sci Policy [Internet]. 2020 Jul;109:116–24. Available from: https://linkinghub.elsevier.com/retrieve/pii/S1462901119314030

[15] Mkhize S, Ellis D. Creativity in marketing communication to overcome barriers to organic produce purchases: The case of a developing nation. J Clean Prod [Internet]. 2020 Jan;242:118415. Available from: https://linkinghub.elsevier.com/retrieve/pii/S0959652619332858

[16] Mesnage R, Tsakiris IN, Antoniou MN, Tsatsakis A. Limitations in the evidential basis supporting health benefits from a decreased exposure to pesticides through organic food consumption. Vol. 19, Current Opinion in Toxicology. 2020. p. 50–5.

[17] Baumann H, Boons F, Bragd A (2002). Mapping the green product development field: Engineering, policy and business perspectives. J Clean Prod.

[18] Mostafidi M, Sanjabi MR, Shirkhan F, Zahedi MT. A review of recent trends in the development of the microbial safety of fruits and vegetables. Vol. 103, Trends in Food Science and Technology. 2020. p. 321–32.

[19] Erhard AL, Chin ER, Chomak ER, Erlendsdottir EY, Perez-Cueto FJA, Orlien V. Exploratory study on purchase intention of vitamin D fortified drinks in Denmark, Iceland, and the UK. Int J Gastron Food Sci [Internet]. 2020 Dec;22:100242. Available from: https://linkinghub.elsevier.com/retrieve/pii/S1878450 × 20301190

[20] Hansmann R, Baur I, Binder CR. Increasing organic food consumption: An integrating model of drivers and barriers. J Clean Prod [Internet]. 2020 Dec;275:123058. Available from: https://linkinghub.elsevier.com/retrieve/pii/S0959652620331036

[21] Shin YH, Jung SE, Im J, Severt K (2020). Applying an extended theory of planned behavior to examine state-branded food product purchase behavior: The moderating effect of gender. J Foodserv Bus Res.

[22] Yazdanpanah M, Forouzani M. Application of the Theory of Planned Behaviour to predict Iranian students’ intention to purchase organic food. J Clean Prod [Internet]. 2015 Nov;107:342–52. Available from: https://linkinghub.elsevier.com/retrieve/pii/S095965261500195X

[23] Yadav R, Pathak GS. Young consumers’ intention towards buying green products in a developing nation: Extending the theory of planned behavior. J Clean Prod [Internet]. 2016 Nov;135:732–9. Available from: https://linkinghub.elsevier.com/retrieve/pii/S0959652616307971

[24] Gao L, Waechter KA. Examining the role of initial trust in user adoption of mobile payment services: an empirical investigation. Inf Syst Front [Internet]. 2017 Jun 23;19(3):525–48. Available from: http://link.springer.com/10.1007/s10796-015-9611-0

[25] Rana J, Paul J (2020). Health motive and the purchase of organic food: A meta-analytic review. Int J Consum Stud.

[26] Icek A (2019). The Theory of Planned Behavior Organizational Behavior and Human Decision Processes. Organ Behav Hum Decis Process.

[27] Ajzen I, Fishbein M. Attitudes and voting behaviour: An application of the theory of reasoned action. In: Progress in applied social psychology. 1981. p. 95–125.

[28] Raza SH, Abu Bakar H, Mohamad B. The effects of advertising appeals on consumers’ behavioural intention towards global brands: The mediating role of attitude and the moderating role of uncertainty avoidance. J Islam Mark [Internet]. 2020 May 17;11(2):449–69. Available from: https://www.emeraldinsight.com/doi/10.1108/JIMA-11-2017-0134

[29] Ajzen I. The theory of planned behavior. Organ Behav Hum Decis Process [Internet]. 1991 Dec;50(2):179–211. Available from: https://linkinghub.elsevier.com/retrieve/pii/074959789190020T

[30] Sniehotta FF, Presseau J, Araújo-Soares V (2014). Time to retire the theory of planned behaviour. Health Psychol Rev.

[31] Canova L, Bobbio A, Manganelli AM. Buying Organic Food Products: The Role of Trust in the Theory of Planned Behavior. Front Psychol. 2020;11. 10.3389/fpsyg.2020.575820 PMC7644777 33192881

[32] Thøgersen J. Consumer decision-making with regard to organic food products. In: Traditional Food Production and Rural Sustainable Development: A European Challenge. 2016. p. 173–92.

[33] Tarkiainen A, Sundqvist S (2005). Subjective norms, attitudes and intentions of Finnish consumers in buying organic food. Br Food J.

[34] Dowd K, Burke KJ. The influence of ethical values and food choice motivations on intentions to purchase sustainably sourced foods. Appetite [Internet]. 2013 Oct;69:137–44. Available from: https://linkinghub.elsevier.com/retrieve/pii/S0195666313002171 10.1016/j.appet.2013.05.024 23770118

[35] Ali A, Sherwani M, Ali A, Ali Z, Sherwani M. Investigating the antecedents of halal brand product purchase intention: an empirical investigation. J Islam Mark [Internet]. 2020 May 22;ahead-of-p(ahead-of-print). Available from: https://www.emerald.com/insight/content/doi/10.1108/JIMA-03-2019-0063/full/html

[36] Smith S, Paladino A (2010). Eating clean and green? Investigating consumer motivations towards the purchase of organic food. Australas Mark J.

[37] Icek A. The Theory of Planned Behavior Organizational Behavior and Human Decision Processes. Organ Behav Hum Decis Process. 2019;50(2).

[38] Sundel M, Sundel SS. Behavior Change in the Human Services: Behavioral and Cognitive Principles and Applications. Behavior Change in the Human Services: Behavioral and Cognitive Principles and Applications. 2019.

[39] Ajzen I (2011). The theory of planned behaviour: Reactions and reflections. Psychol Health.

[40] Dagher GK, Itani O (2014). Factors influencing green purchasing behaviour: Empirical evidence from the Lebanese consumers. J Consum Behav.

[41] Spears N, Singh SN (2004). Measuring attitude toward the brand and purchase intentions. J Curr Issues Res Advert.

[42] Hong KJ, Park NL, Heo SY, Jung SH, Lee YB, Hwang JH. Effect of e-Health Literacy on COVID-19 Infection-Preventive Behaviors of Undergraduate Students Majoring in Healthcare. Healthcare [Internet]. 2021 May 12;9(5):573. Available from: https://www.mdpi.com/2227-9032/9/5/573 10.3390/healthcare9050573 PMC8151528 34066120

[43] Schifferstein HNJ, Oude Ophuis PAM. Health-related determinants of organic food consumption in the Netherlands. Food Qual Prefer. 1998;9(3).

[44] Chen MF (2011). The joint moderating effect of health consciousness and healthy lifestyle on consumers’ willingness to use functional foods in Taiwan. Appetite.

[45] Michaelidou N, Hassan LM (2008). The role of health consciousness, food safety concern and ethical identity on attitudes and intentions towards organic food. Int J Consum Stud.

[46] Forthofer MS, Bryant CA (2000). Using audience-segmentation techniques to tailor health behavior change strategies. Am J Health Behav.

[47] Lim CGY, van Dam RM. Attitudes and beliefs regarding food in a multi-ethnic Asian population and their association with socio-demographic variables and healthy eating intentions. Appetite [Internet]. 2020 Jan;144:104461. Available from: https://linkinghub.elsevier.com/retrieve/pii/S0195666319306956 10.1016/j.appet.2019.104461 31539580

[48] Chen MF. Influences of health consciousness on consumers’ modern health worries and willingness to use functional foods. J Appl Soc Psychol [Internet]. 2013 Jun;43(SUPPL.1):E1–12. Available from: https://doi.org/doi.wiley.com/10.1111/jasp.12033

[49] Landström E, Koivisto Hursti UK, Becker W, Magnusson M (2007). Use of functional foods among Swedish consumers is related to health-consciousness and perceived effect. Br J Nutr.

[50] Kaygusuz K. Energy and environmental issues relating to greenhouse gas emissions for sustainable development in Turkey. Vol. 13, Renewable and Sustainable Energy Reviews. 2009. p. 253–70.

[51] Alzubaidi H, Slade EL, Dwivedi YK (2021). Examining antecedents of consumers’ pro-environmental behaviours: TPB extended with materialism and innovativeness. J Bus Res.

[52] Lee K (2008). Opportunities for green marketing: Young consumers. Mark Intell Plan.

[53] Yarimoglu E, Gunay T (2020). The extended theory of planned behavior in Turkish customers’ intentions to visit green hotels. Bus Strateg Environ.

[54] Yin S, Wu L, Du L, Chen M. Consumers’ purchase intention of organic food in China. J Sci Food Agric. 2010;90(8). 10.1002/jsfa.3936 20474056

[55] Hien NN, Phuong NN, van Tran T, Thang LD (2020). The effect of country-of-origin image on purchase intention: The mediating role of brand image and brand evaluation. Manag Sci Lett.

[56] Cotte J, Wood SL (2004). Families and innovative consumer behavior: A triadic analysis of sibling and parental influence. J Consum Res.

[57] Zhang F, Sun S, Liu C, Chang V. Consumer innovativeness, product innovation and smart toys. Electron Commer Res Appl [Internet]. 2020 May;41:100974. Available from: https://linkinghub.elsevier.com/retrieve/pii/S156742232030051X

[58] Midgley DF, Dowling GR. Innovativeness: The Concept and Its Measurement. J Consum Res [Internet]. 1978 Mar;4(4):229. Available from: https://academic.oup.com/jcr/article-lookup/doi/10.1086/208701

[59] Venkatraman MP (1991). The impact of innovativeness and innovation type on adoption. Journal of Retailing.

[60] Persaud A, Schillo SR (2017). Purchasing organic products: role of social context and consumer innovativeness. Mark Intell Plan.

[61] Azzurra A, Massimiliano A, Angela M (2019). Measuring sustainable food consumption: A case study on organic food. Sustain Prod Consum.

[62] Goetzke B, Nitzko S, Spiller A. Consumption of organic and functional food. A matter of well-being and health? Appetite [Internet]. 2014 Jun;77:96–105. Available from: https://linkinghub.elsevier.com/retrieve/pii/S0195666314001068 10.1016/j.appet.2014.02.012 24630940

[63] Bloch PH (1984). The wellness movement: Imperatives for health care marketers. Journal of Health Care Marketing.

[64] Güney OI, Giraldo L (2019). Consumers’ attitudes and willingness to pay for organic eggs: A discrete choice experiment study in Turkey. Br Food J.

[65] Fraj E, Martinez E. Environmental values and lifestyles as determining factors of ecological consumer behaviour: an empirical analysis. J Consum Mark [Internet]. 2006 Apr;23(3):133–44. Available from: https://www.emerald.com/insight/content/doi/10.1108/07363760610663295/full/html

[66] Zarif Sagheb M, Ghasemi B, Nourbakhsh SK (2020). Factors affecting purchase intention of foreign food products: An empirical study in the Iranian context. Br Food J.

[67] Gong W. Effects of parasocial interaction, brand credibility and product involvement on celebrity endorsement on microblog. Asia Pacific J Mark Logist [Internet]. 2020 Nov 25;ahead-of-p(ahead-of-print). Available from: https://www.emerald.com/insight/content/doi/10.1108/APJML-12-2019-0747/full/html

[68] Erdem H, Şimşek I, Pay S, Dinc A, Deniz O, Ozcan A (2006). Diffuse pulmonary amyloidosis that mimics interstitial lung disease in a patient with familial mediterranean fever. J Clin Rheumatol.

[69] Martín-Consuegra D, Faraoni M, Díaz E, Ranfagni S (2018). Exploring relationships among brand credibility, purchase intention and social media for fashion brands: A conditional mediation model. J Glob Fash Mark.

[70] Jun S-H. The Effects of Perceived Risk, Brand Credibility and Past Experience on Purchase Intention in the Airbnb Context. Sustainability [Internet]. 2020 Jun 26;12(12):5212. Available from: https://www.mdpi.com/2071-1050/12/12/5212

[71] Spry A, Pappu R, Cornwell TB (2011). Celebrity endorsement, brand credibility and brand equity. Eur J Mark.

[72] Konuk FA. Price fairness, satisfaction, and trust as antecedents of purchase intentions towards organic food. J Consum Behav. 2018;17(2).

[73] Skiba T, Wildman JL. Uncertainty Reducer, Exchange Deepener, or Self-Determination Enhancer? Feeling Trust Versus Feeling Trusted in Supervisor-Subordinate Relationships. J Bus Psychol [Internet]. 2019 Apr 23;34(2):219–35. Available from: http://link.springer.com/10.1007/s10869-018-9537-x

[74] Lassoued R, Hobbs JE. Consumer confidence in credence attributes: The role of brand trust. Food Policy. 2015;52.

[75] Ngo HM, Liu R, Moritaka M, Fukuda S (2020). Effects of industry-level factors, brand credibility and brand reputation on brand trust in safe food: evidence from the safe vegetable sector in Vietnam. Br Food J.

[76] de Jonge J, van Trijp H, Goddard E, Frewer L. Consumer confidence in the safety of food in Canada and the Netherlands: The validation of a generic framework. Food Qual Prefer. 2008;19(5).

[77] Ansary A, Nik Hashim NMH (2018). Brand image and equity: the mediating role of brand equity drivers and moderating effects of product type and word of mouth. Rev Manag Sci.

[78] Devedi P, Sujatha R, Pathak R (2017). A study on parameters of online reviews content that influence consumers buying behaviour- an Indian perspective. J Bus Retail Manag Res.

[79] Plumeyer A, Kottemann P, Böger D, Decker R. Measuring brand image: a systematic review, practical guidance, and future research directions. Vol. 13, Review of Managerial Science. 2019. p. 227–65.

[80] Li H, Lo H-Y. Do You Recognize Its Brand? The Effectiveness of Online In-Stream Video Advertisements. J Advert [Internet]. 2015;44(3). Available from: http://www.tandfonline.com/doi/abs/10.1080/00913367.2014.956376

[81] Alamsyah DP, Othman NA, Mohammed HAA (2020). The awareness of environmentally friendly products: The impact of green advertising and green brand image. Manag Sci Lett.

[82] Durmaz Y, Cavusoglu S, Ozer O. The Effect of Brand Image and Brand Benefit on Customer Loyalty: The Case of Turkey. Int J Acad Res Bus Soc Sci [Internet]. 2018 Jun 4;8(5):528–40. Available from: http://hrmars.com/index.php/journals/papers/IJARBSS/v8-i5/4140

[83] Devedi P. A study on parameters of online reviews content that influence consumers buying behaviour- an Indian perspective. J Bus Retail Manag Res [Internet]. 2017;11(4):12–24. Available from: 10.24052/jbrmr/v11is04/asopoorcticcbbaap/pd/rs/rp

[84] Kajale DB, Becker TC (2015). Factors Influencing Young Consumers’ Acceptance of Genetically Modified Food in India. J Food Prod Mark.

[85] Jäger A-K, Weber A. Can you believe it? The effects of benefit type versus construal level on advertisement credibility and purchase intention for organic food. J Clean Prod [Internet]. 2020 Jun;257:120543. Available from: https://linkinghub.elsevier.com/retrieve/pii/S0959652620305904

[86] Raza SH, Zaman U, Ferreira P, Farías P. An Experimental Evidence on Public Acceptance of Genetically Modified Food through Advertisement Framing on Health and Environmental Benefits, Objective Knowledge, and Risk Reduction. Int J Environ Res Public Health [Internet]. 2021 May 15 [cited 2021 May 16];18(10):5264. Available from: https://www.mdpi.com/1110356 10.3390/ijerph18105264 PMC8156498 34063370

[87] Hingston ST, Noseworthy TJ (2018). Why consumers don’t see the benefits of genetically modified foods, and what marketers can do about it. J Mark.

[88] Adeyeye SAO, Idowu-Adebayo F (2019). Genetically modified and biofortified crops and food security in developing countries: A review. Nutr Food Sci.

[89] Facebook Inc. Facebook Reports First Quarter 2019 Results. Invest Relations [Internet]. 2019;1. Available from: https://investor.fb.com/investor-news/press-release-details/2019/Facebook-Reports-First-Quarter-2019-Results/default.aspx

[90] Chin WW. Issues and opinion on structural equation modeling. Vol. 22, MIS Quarterly: Management Information Systems. 1998. p. 12–6.

[91] Fornell C, Larcker DF. Evaluating Structural Equation Models with Unobservable Variables and Measurement Error. J Mark Res [Internet]. 1981 Feb;18(1):39. Available from: http://www.jstor.org/stable/3151312?origin=crossref

[92] Henseler J, Hubona G, Ray PA (2016). Using PLS path modeling in new technology research: updated guidelines. Ind Manag Data Syst.

[93] Leguina A (2015). A primer on partial least squares structural equation modeling (PLS-SEM). Int J Res Method Educ.

[94] Geisser S (1974). A predictive approach to the random effect model. Biometrika.

[95] Ganjoo M. Influence of advertising appeals on buying behaviour with reference to cosmetic brands. Int J Psychosoc Rehabil [Internet]. 2020;24(8):8222–9. Available from: https://www.scopus.com/inward/record.uri?eid=2-s2.0-85088707318&doi=10.37200%2FIJPR%2FV24I8%2FPR280831&partnerID=40&md5=d48f334c5550e46817747d16964cc353

[96] Ajzen I (1991). The theory of planned behavior. Organ Behav Hum Decis Process.

[97] Dang HP, Nguyen Viet B. Inside the intention to join extracurricular activities: Integrating the theory of planned behavior and signaling theory. Cogent Educ. 2021;8(1).

[98] Connelly BL, Certo ST, Ireland RD, Reutzel CR (2011). Signaling theory: A review and assessment. Journal of Management.

[99] Özsomer A, Altaras S. Global brand purchase likelihood: A critical synthesis and an integrated conceptual framework. J Int Mark. 2008;16(4).

[100] Fischer-Preßler D, Schwemmer C, Fischbach K. Collective sense-making in times of crisis: Connecting terror management theory with Twitter user reactions to the Berlin terrorist attack. Comput Human Behav [Internet]. 2019 Nov;100:138–51. Available from: https://linkinghub.elsevier.com/retrieve/pii/S0747563219301876

[101] Abdullah A, Yaacob MR, Ismail M, Zakaria MN, Abdullah Z, Mohd Radyi SA (2017). Corporate engagement with the community: Building relationships through CSR. J Eng Appl Sci.

[102] Abdullah Z (2009). Beyond corporate image: Projecting international reputation management as a new theoretical approach in a transitional country. Int J Econ Manag.

[103] Abdullah Z (2007). Towards international cultural diversity management of public relations: Viewpoints of chairmen/CEOs. Int J Econ Manag.

[104] Abdullah Z. Cultural diversity management in Malaysia: A perspective of communication management. In: Managing Cultural Diversity in Asia: A Research Companion. 2010. p. 14–38.

[105] Sosoo EE, Bernard DL, Neblett EW. The influence of internalized racism on the relationship between discrimination and anxiety. Cult Divers Ethn Minor Psychol [Internet]. 2020 Oct;26(4):570–80. Available from: http://doi.apa.org/getdoi.cfm?doi=10.1037/cdp0000320 10.1037/cdp0000320 PMC7359777 31886684

